# Tuberculoma of the liver

**DOI:** 10.4103/0971-9261.44769

**Published:** 2008

**Authors:** A. Bharathi, K. Nagarjuna, G. V. S. N. Prasad, J. Bhaskar Reddy, D. Kalyan Ravi Prasad

**Affiliations:** Department of Professor of Paediatric Surgery, Niloufer Institute of Child Health, Hyderabad, India; 1 Asst. professor, Niloufer Institute of Child Health, Hyderabad, India

**Keywords:** Liver mass, tuberculoma, tuberculosis

## Abstract

We report an isolated giant solid macronodular tuberculoma in an 8-year-old boy. A large-space-occupying lesion in the right lobe with nodular surface and hard consistency mimicked liver malignancy. This case is unusual as the ultrasonography, computed tomography scan, and aspiration cytology were all suggestive of a malignant tumor. Laparotomy confirmed a 15 × 10 cm nodular tumor present in the right lobe of liver. The segments 5, 6, 7, and 8 were excised. The histopathology revealed tuberculosis.

## INTRODUCTION

Liver is a rare site for tuberculosis.[1] Isolated giant solid macronodular tuberculoma of liver[2,3] is even rarer. Only a few cases are described in the literature and in several of these, the tumor is of small size and not isolated. This rare localization of extra pulmonary tuberculosis presented with a solid inflammatory lesion with a tumor-like appearance. We report a case of isolated giant solid macronodular tuberculoma.

## CASE REPORT

**Figure 1:**
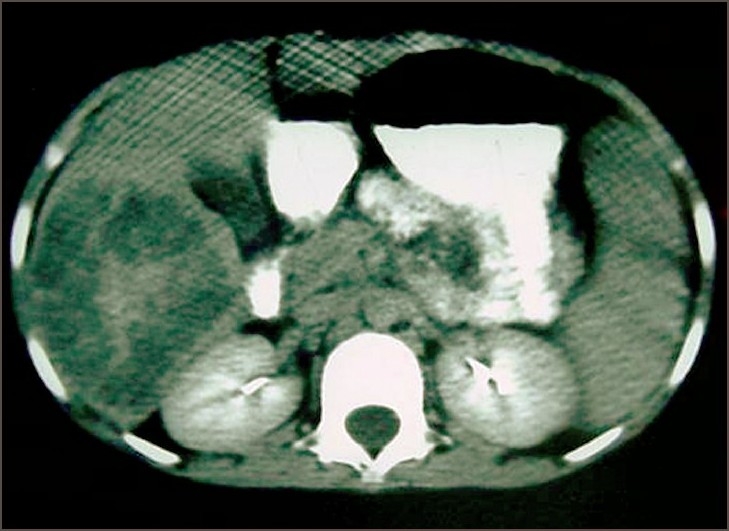
CT scan shows large irregular solid heterogenous enhancing mass in the right lobe of liver

An 8-year-old boy presented with history of intermittent low-grade fever, and loss of appetite and weight of six months duration. The child was immunized with BCG vaccine. The child had protein energy malnutrition with nontender hepatomegaly. The laboratory findings showed Hb, 9 gm%; TLC, 16,800/cu mm; DLC of neutrophils, 83%, lymphocytes, 12%, eosinophils, 2%, monocytes, 1%; and the ESR, 10 mm. The renal and liver functions were normal. Montoux skin test showed 15 mm induration. The chest and abdomen X-rays were unremarkable. Ultrasound examination showed a hyperechoic solitary 15 × 15 cm lesion occupying the right hepatic lobe. Contrast enhanced computed tomography (CECT) scans [Figure 1] revealed a large irregular solid heterogenous enhancing mass present in the right lobe of liver suggestive of malignancy. CT guided fine needle aspiration cytology (FNAC) on two separate occasions suggested hepatoblastoma. Among the tumor markers, AFP was 1.6 ng/ml and beta HCG was< 10 miu/ml.

**Figure 2:**
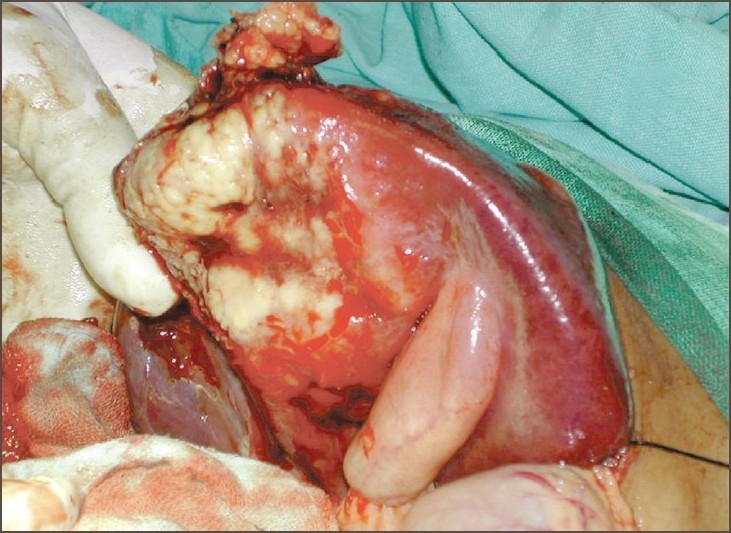
Operative picture shows large nodular tumor present in the right lobe of liver

The abdomen was explored by a roof-top skin incision. A 15 × 10 cm size nodular tumor was seen involving the entire anatomical right lobe of the liver [Figure 2]. There was no ascites. The peritoneum and other intra-abdominal organs were normal. Aspiration of the lesion revealed blood. With a pre and intraoperative diagnosis of malignant hepatic tumor, right hepatectomy was done with resection of segment 5, 6, 7, and 8. The postoperative period was uneventful.

Macroscopically, a large mass of 13 × 10 × 10 cm size with solid areas and foci of necrosis was seen replacing the normal liver tissue. In addition, an 8 × 5 cm size tumor tissue was infiltrating the capsule and forming nodular surface. The gallbladder was seen adherent to the undersurface with 1 cm wall thickness. The cut surface was homogenously white. The histology revealed large areas of caseous necrosis and clusters of epitheliod cells and scattered Langerhan's giant cells. Polymerase chain reaction (PCR) analysis of tumor tissue was positive for Mycobacterium tuberculosis. The patient was subsequently treated with antitubercular therapy and is under follow-up.

## DISCUSSION

Tuberculosis of liver is uncommon[1] and it includes miliary tuberculosis, pulmonary tuberculosis with liver involvement, primary liver tuberculosis, tuberculoma (abscess), and tuberculous cholangitis.[4] Among these, liver tuberculosis is primarily due to miliary infection.[5] Tuberculoma,[6–12] a solid inflammatory lesion of tuberculosis, is rare and isolated giant macronodular tuberculoma is rarer.

Hepatic macronodular tuberculoma is not uncommonly misdiagnosed as a liver tumor. On ultrasonography, the tuberculoma presented in our case as a hyperechoic lesion in contrast to round hypoechoic mass which is usually seen in this condition. The CECT showed the lesion replacing the entire anatomical right lobe with enhancement resembling an infiltrative tumor. To add to the confusion, FNAC was suggestive of hepatoblastoma.

The main stay of treatment is antitubercular drug therapy. However in the literature, hepatectomy has been done in some cases[6,7] as a part of effective treatment along with antitubercular therapy or when mistakenly diagnosed as malignancy or for complicated tuberculoma. Surgical enucleation of the mass apart from full antitubercular therapy may be required as the large mass, unlike brain tuberculoma, would not dissolve with drugs.[10] Prochazka *et al*,[9] treated a hepatic tuberculoma complicated by bleeding with extended right lobectomy.

Isolated giant solid macronodular tuberculoma has a pseudotumor appearance and mimics malignant primary or secondary liver tumors. Keeping in view the large size of mass and extra pulmonary localization, surgical enucleation along with antitubercular drug therapy could be more effective in the treatment of such cases.
